# Neuroaxonal Injury May Mediate the Association Between Hyperglycemia and Prognosis in Spontaneous Subarachnoid Hemorrhage

**DOI:** 10.1007/s12035-024-04347-6

**Published:** 2024-07-12

**Authors:** Daniel Santana, Laura Llull, Alejandra Mosteiro, Leire Pedrosa, Gabriel Pujol, Luigi Zattera, Mariano Werner, Abraham Martín, Carles Justicia, Ángel Chamorro, Ramón Torné, Sergio Amaro

**Affiliations:** 1 Institute of Neuroscience, Comprehensive Stroke Center, Hospital Clinic of Barcelona; University of Barcelona, Barcelona, Spain; 2 Institute of Neuroscience, Neurosurgery Department, Hospital Clinic of Barcelona; University of Barcelona, Barcelona, Spain; 3 Institut d’Investigacions Biomèdiques Agustí Pi I Sunyer (IDIBAPS), Barcelona, Spain; 4 Neuroanesthesia Division, Anesthesiology Department, Hospital Clinic of Barcelona, Barcelona, Spain; 5 Neurocritical Care Division, Anthesiology Department, Hospital Clinic of Barcelona, Barcelona, Spain; 6 Institute of Diagnostic Imaging, Interventional Neurorradiology Department, Hospital Clinic of Barcelona, Barcelona, Spain; 7 Achucarro Basque Center for Neuroscience, Leioa, Spain; 8 Ikerbasque Basque Foundation for Science, Bilbao, Spain; 9 Instituto de Investigaciones Biomédicas de Barcelona (IIBB), Consejo Superior de Investigaciones Científicas (CSIC), Barcelona, Spain

**Keywords:** Subarachnoid hemorrhage, Early brain injury, Neurofilament, Glucose, Neuroaxonal damage, Mediation analysis

## Abstract

Hyperglycemia during early brain injury (EBI) period after spontaneous subarachnoid hemorrhage (SAH) is associated with poor outcome, but the underlying physiopathology is unknown. This study assessed if hyperglycemia during EBI is associated with markers of neuroaxonal injury and whether these biomarkers partially account for the association between hyperglycemia and poor clinical outcome. Ninety-two SAH patients admitted within 24 h of bleeding onset were prospectively included. Glucose levels were measured at arrival and every 6 h for 72 h. Serum neurofilament light chain (NFL) levels were measured at 72 h. Functional outcome was assessed with the modified Rankin Scale (mRS) at 90 days (poor outcome, mRS > 2). The association between glucose metrics, NFL levels, and clinical outcome was assessed with univariate and multivariate analyses. Mediation analysis was performed to examine the potential chain in which NFL may mediate the relationship between glucose and functional outcome. Higher glucose and NFL levels during EBI were associated with poor clinical outcome in adjusted analysis. NFL levels were associated with older age, higher initial severity, and higher glucose levels during EBI period. In adjusted mediation analyses, the association between glucose and clinical outcome was significantly mediated by NFL levels. The mediator NFL explained 25% of the association between glucose during EBI period and poor functional outcome at 90 days. In SAH, the association between glucose levels during EBI and poor clinical outcome might be significantly mediated by NFL levels. The link between hyperglycemia and poor clinical outcome might be explained in part through secondary neuroaxonal injury.

**Supplementary Information:**

The online version contains supplementary material available at 10.1007/s12035-024-04347-6.

## Introduction

Spontaneous subarachnoid hemorrhage (SAH) is a devastating cerebrovascular disease caused by the rupture of an intracranial aneurysm in 85% of cases [1, 2]. The disability burden caused by SAH is partly driven by several harmful mechanisms triggered within the first 72 h after bleeding onset resulting in the so-called early brain injury (EBI), which has been consistently associated with an increased risk of delayed systemic complications as well as long-term poor outcome [3, 4].

The mechanisms implicated in EBI are still partly known and include microthrombosis, oxidative stress, blood–brain barrier disruption, and multifocal axonal injury, among others [5–10]. In acute central nervous system diseases including brain hemorrhage, most of these deleterious mechanisms are enhanced after the exposition to high glucose levels [11–14]. Hyperglycemia occurs in about three out of four SAH patients within the EBI period, and it has been associated with poor clinical outcome [15–20]. Hyperglycemia is in part a consequence of an acute stress response to brain injury and enhances a myriad of deleterious physiopathological processes including neuroinflammatory mechanisms that eventually may promote diffuse neuroaxonal damage (NAD) [21–23]. However, there are no reports on the link between abnormal glucose profiles during the EBI period and NAD biomarkers.

Neurofilament light chains (NFL) are structural proteins mainly present in the cytoskeleton of myelinated neurons. Following axonal injury, NFLs are released to the extracellular compartment hence being considered surrogate biomarkers of NAD [24]. In the setting of SAH, NFL levels measured in blood and cerebrospinal fluid samples collected during the EBI period as biomarkers of ongoing brain injury are strongly correlated with poor clinical outcome metrics at short and long term [25–33]. These observations suggest that NAD could play a major role in the EBI phase after SAH, although the mechanisms implicated in the severity of NAD remain to be fully understood.

Herein, we hypothesized that in SAH sufferers, the prognostic relevance of hyperglycemia during the EBI period could be at least in part mediated through its relationship with NAD. Thus, our specific objective was to evaluate in a prospective cohort of SAH subjects whether the association between hyperglycemia and poor clinical outcome was mediated by diffuse NAD as measured with circulating NFL levels at the end of the EBI period. Eventually, determining the link between glucose dysregulations during the EBI period, NAD and long-term clinical outcome could be of interest for designing and monitoring the effects of new interventions in clinical trials aimed to modulate glucose levels or the mechanisms associated with glucose-driven secondary brain injury.

## Methods

### Study Design and Population

We conducted an observational, single-center, prospective cohort study in a tertiary level referral hospital (> 50 SAH admissions per year) provided with a multidisciplinary Comprehensive Stroke Center, a Stroke Unit and an Intensive Care Unit specialized in neurocritical care. Participants were included in the study from September 2018 to June 2021. The study inclusion criteria were age older than 18 years, good premorbid functional status [modified Rankin Scale (mRS) 0–2], and spontaneous SAH admitted at our center in the first 24 h from symptom onset. Exclusion criteria included the presence of a non-aneurysmal exclusively perimesencephalic blood pattern in the admission CT, previous history of diabetes mellitus, SAH secondary to other causes (i.e., traumatic, venous thrombosis, mycotic aneurysms), or very short life expectancy that could compromise follow-up. A schematic flowchart of the study is shown in Supplementary Fig. 1. Ethics approval was obtained from the local Clinical Research Ethics Committee from Hospital Clinic of Barcelona (HCB/2017/027). Patients or a legal proxy gave written informed consent prior to inclusion in the study.

### Clinical Assessment

Baseline demographic data, past medical history, clinical features at SAH onset, and neuroimaging results were prospectively recorded. Neurological condition on admission was assessed according to the World Federation of Neurosurgical Societies (WFNS) grading system after initial resuscitation. Patients were grouped into good grade (WFNS grades 1 and 2) or poor grade (WFNS grades 3 to 5) SAH. Patients with SAH were admitted to the critical care unit (WFNS 3–5) or the Stroke Unit (WFNS 1–2) as clinically appropriate. Neurological status [Glasgow Coma Scale (GCS) and National Institutes Health Stroke Scale (NIHSS)] were assessed daily by neurologists in the Stroke Unit or anesthesiologists in the critical care unit. Systemic and neurological complications were monitored and treated according to in-hospital protocols.

GCS is a widely used assessment tool of patients’ level of consciousness, based on three items: eye opening, verbal response, and motor response. It ranges from 15 (full consciousness) to 3 (deep comma) [34]. WFNS is a SAH-specific grading system that classifies patients into five groups, according to their GCS and presence or absence of motor deficit [35]. Thus, WFNS I represents a SAH patient with unpaired consciousness (GCS 15) and no motor deficit, whereas WFNS V represents a comatose patient (GCS 3 to 6). Lastly, NIHSS is a standardized assessment tool mainly used in the context of cerebrovascular diseases to evaluate focal deficits in several neurological fields such as level of consciousness, speech, motor, and sensory functions. It ranges from 0 (no deficit) to 42 (maximum deficit) and allows a precise and reproducible monitoring of the extent of brain damage [36]. All three scales have been widely reported in medical literature in the context of SAH, with significant associations between worse scores and poorer prognosis.

### Imaging Methods

Non-contrast brain CT was performed to all patients at arrival. The extent of subarachnoid bleeding was categorized according to the modified Fisher Scale (mF) classification. This scale categorizes the amount of blood observed in non-contrast brain CT into four groups, according to bleeding thickness in subarachnoid space and the presence or absence of intraventricular blood clots. It ranges from 1 (thin subarachnoid bleeding, no intraventricular blood) to 4 (thick subarachnoid bleeding, intraventricular blood) and is positively correlated with the risk and severity of complications associated with the bleeding, mainly arterial vasospasm [37]. Additional relevant radiological features including the presence of intraventricular hemorrhage, hydrocephalus, or intraparenchymal hematoma were also collected. Additional imaging acquisitions were performed at discretion of the attending physician according to clinical evolution. An Angio-CT was performed to all patients at arrival, as well as a cerebral angiography within the first 24 h after hospital admission, as part of vascular study. According to the presence or absence of a brain aneurysm, the patients were further classified as non-aneurysmal SAH or aneurysmal SAH. In the latter group, data on the number, location, size, and anatomical features of aneurysms were recorded, and the index aneurysm was evaluated for endovascular or surgical exclusion treatment according to in-hospital protocols.

### Sampling and Laboratory Analysis

Blood glucose was measured through finger prick testing at arrival and every 6 h for at least the following 72 h, as part of usual clinical practice. Glucose management consisted on a sliding scale in fasting patients and/or patients receiving continuous nutrition through a nasogastric tube and a basal-bolus insulin therapy in patients receiving regular meals. Maximum, minimum, and mean glucose levels during the first 72 h were recorded.

To evaluate serum NFL levels, an additional blood sample (15 mL) was drawn at 72 h after hospital admission. The samples were centrifuged at 4000 rpm (1523 relative centrifugal force) for 15 min at 20–22 °C, and supernatant was pipetted and frozen at − 80 °C until further analysis. NFL levels were measured through the Simoa technique (Quanterix, Lexington, MA, USA) in serum samples. In brief, Simoa is a fourth-generation ELISA based on proteic antigen capturing by paramagnetic microscopic beads that carry sandwich antibody complexes. Then, those beads are precipitated into single-molecule arrays that are individually read to check for their bond to the index antigen. Batch serum NFL measurement was performed according to the manufacturer’s guide.

### Clinical Outcome Evaluation

The predefined primary outcome measure was functional disability, evaluated with the modified Rankin Scale (mRS). Functional outcome was assessed by certified neurologists on in-person visits at the outpatient clinic 90 days after initial hospital admission, through a semi-structured clinical interview. Poor functional outcome was predefined as mRS > 2.

### Statistical Analysis

Continuous variables are shown as mean (standard deviation) or median (interquartile intervals), and differences between groups were assessed using Student’s *t*-test, ANOVA, or Mann–Whitney as appropriate. Categorical variables are shown as absolute values (percentage) and compared using chi-squared or Fisher’s exact tests. A univariate analysis was run to assess the association of demographic, clinical, and treatment-related variables with clinical outcome at 3 months and with NFL levels. The accuracy of the explored glucose metrics for predicting clinical outcome was assessed with receiver operating characteristic curves (ROC), and the glucose metric with higher area under the curve (AUC) was selected for its inclusion in multivariate models. A multivariate logistic regression model analyzed the predictors of poor clinical outcome, and a multivariate linear regression assessed the variables associated with NFL levels. Spearman’s correlation analyses were performed to assess the associations between glucose metrics, NFL levels, and mRS scores, and the numerical results were illustrated through a correlation matrix. For linear regression and mediation analyses, we used log-transformed NFL levels to approach normality. Age and WFNS at admission were forced to remain in the final model for their prognostic relevance based on prior knowledge. To avoid model overfitting, a backward elimination method (likelihood ratio) was used to select the final binary logistic regression model, and the Hosmer–Lemeshow goodness-of-fit statistic was used to assess the final model fit. Mediation analyses were also implemented to assess whether glucose influenced clinical outcome indirectly via NFL by using the PROCESS macro (Hayes, 2013) for the statistical software IBM SPSS Statistics for Windows (IBM, Armonk, NY, USA); 10,000 bootstraps produced 95% confidence intervals (CI) on the mediation coefficient parameter estimates, and mediated (indirect) effects were considered significant if the 95% CI for their parameter estimates did not cross the zero value. The analyses were performed using SPSS version 25.0, and the level of significance was established at a 0.05 level (two-sided).

### Data Availability

The datasets generated and analyzed are available from the corresponding authors on reasonable request.

## Results

### Characteristics of the Study Population

A total of 92 patients were included during the study period. The main traits of the included patients are shown in Table 1. Overall, 61 (66%) patients were women with a median (IQR) age of 55 (49–65) years. On admission, 36 (39%) patients presented with poor clinical grade SAH (WFNS 4–5), and 67 (73%) had a modified Fisher Scale score of 4. A brain aneurysm was found in 84 (91%) patients, from whom 57 (62%) were treated endovascularly and 23 (25%) received surgical clipping. Initial neuroimaging assessment revealed the presence of hydrocephalus in 58 (63%) patients, parenchymal hemorrhage in 22 (24%), and intraventricular bleeding in 68 (74%).

**Table 1:** Study population characteristics assessed during the early brain injury period according to clinical outcome at 3 months (whole sample)

	mRS 0–2 (*n* = 60)	mRS 3–6 (*n* = 32)	*p*
Demographics			
Age (years)	54 (49–62)	61 (52–74)	0.054
Female sex	39 (65)	22 (69)	0.717
Pre-morbid mRS	0 (0–0)	0 (0–1)	0.003
Medical history			
Hypertension	21 (35)	17 (53)	0.093
Dyslipidemia	9 (15)	8 (25)	0.239
Baseline traits			
WFNS grade (4–5)	13 (22)	23 (72)	< 0.001
Aneurysm (yes)	54 (90)	30 (94)	0.543
Aneurysm size (mm)	5 (4–6)	6 (5–9)	0.010
Modified Fisher Scale	4 (3–4)	4 (4–4)	0.033
Intraventricular bleeding	41 (68)	27 (84)	0.095
Hydrocephalus	33 (55)	25 (68)	0.029
Parenchymal hemorrhage	13 (22)	9 (28)	0.489
Global cerebral edema	33 (55)	20 (63)	0.488
Aneurysm treatment modality			0.596
No	8 (13)	4 (13)	
Endovascular	39 (65)	18 (56)	
Surgical	13 (22)	10 (31)	
Glucose metrics			
Glucose at admission (mg/dL)	125 (113–142)	138 (126–164)	0.005
Mean glucose within 72 h (mg/dL)	114 (103–128)	137 (123–150)	< 0.001
Max glucose within 72 h (mg/dL)	150 (126–164)	165 (150–188)	0.001
Min glucose within 72 h (mg/dL)	87 (81–100)	103 (97–121)	< 0.001
SD glucose within 72 h (mg/dL)	18 (14–24)	23 (16–28)	0.106

Qualitative data are expressed as *n* (%) and quantitative data as median (IQR)

*mRS* modified Rankin Scale, *SD* standard deviation

### Glucose Metrics During the EBI Period

Glucose metrics during the EBI period including glucose at admission, maximum, minimum, mean, and standard deviation of glucose levels are shown in Table 1. In univariate analyses, all glucose metrics except the standard deviation (SD) were associated with clinical outcome at 90 days. In binary logistic regression multivariate analyses adjusted by baseline clinical severity (WFNS), only mean glucose levels and minimum glucose levels within the first 72 h after bleeding onset remained associated with poor clinical outcome at 90 days [mean glucose levels, OR (95% CI) per each mg/dL of increase 1.04 (1.010–1.077), *p* = 0.010; minimum glucose levels, OR (95% CI) per each mg/dL of increase 1.04 (1.007–1.079), *p* = 0.020]. In agreement with these observations, in ROC analysis, mean glucose levels during the EBI period disclosed the highest AUC value, followed by minimum glucose during the EBI period, maximum glucose during the EBI period, and glucose levels at hospital admission, as shown in Table 2. According to these findings, mean glucose levels during the first 72 h after hospital admission were used for further analyses.

**Table 2:** Receiver operating characteristic curve analyses of the accuracy of glucose metrics for predicting clinical outcome at 90 days

Glucose metrics	AUC (95% CI), *p*	*p* value
Glucose at admission	0.680 (0.569–0.792)	0.005
Mean glucose within 72 h	0.797 (0.706–0.889)	< 0.001
Max glucose within 72 h	0.708 (0.600–0.817)	0.001
Min glucose within 72 h	0.764 (0.664–0.863)	< 0.001
SD glucose within 72 h	0.605 (0.481–0.729)	0.098

*AUC* area under the curve, *CI* confidence interval, *SD* standard deviation

### Variables Associated with Poor Functional Outcome

In this cohort, a total of 32 (35%) patients showed poor functional outcome at 90 days. In univariate analyses, poor outcome was associated with worse premorbid mRS, poorer initial WFNS grade, larger aneurysm size, higher mF score, and hydrocephalus at initial neuroimaging, as well as with higher glucose levels during the first 72 h (Table 1). In multivariate models, the variables that remained independently associated with poor clinical outcome were poorer WFNS grade at onset and higher mean glucose levels during the EBI period (Table 3).

**Table 3:** Variables assessed during EBI period associated with poor clinical outcome (mRS > 2): multivariate analysis

Variables	OR (95% CI)	*p* value
Age (per year)	1.02 (0.980–1.069)	0.303
Pre-morbid mRS (per IQR)	1.90 (0.932–3.865)	0.077
WFNS grade (4–5)	4.05 (1.276–12.877)	0.018
Mean glucose within 72 h (per mg/dL)	1.04 (1.009–1.081)	0.014

The variables that remained in the final model were selected through binary logistic regression with backward procedure (likelihood ratio). The initial model included all the variables showing a *p* value of < 0.1 in univariate analysis (shown in Table 1). Age and WFNS at admission were forced to remain in the final model

*OR* odds ratio, *mRS* modified Rankin Scale, *IQR* interquartile range, *WFNS* World Federation of Neurosurgical Societies Scale

### Circulating NFL Levels at the End of the EBI Period: Contributors and Association with Clinical Outcome

**Fig. 1:**
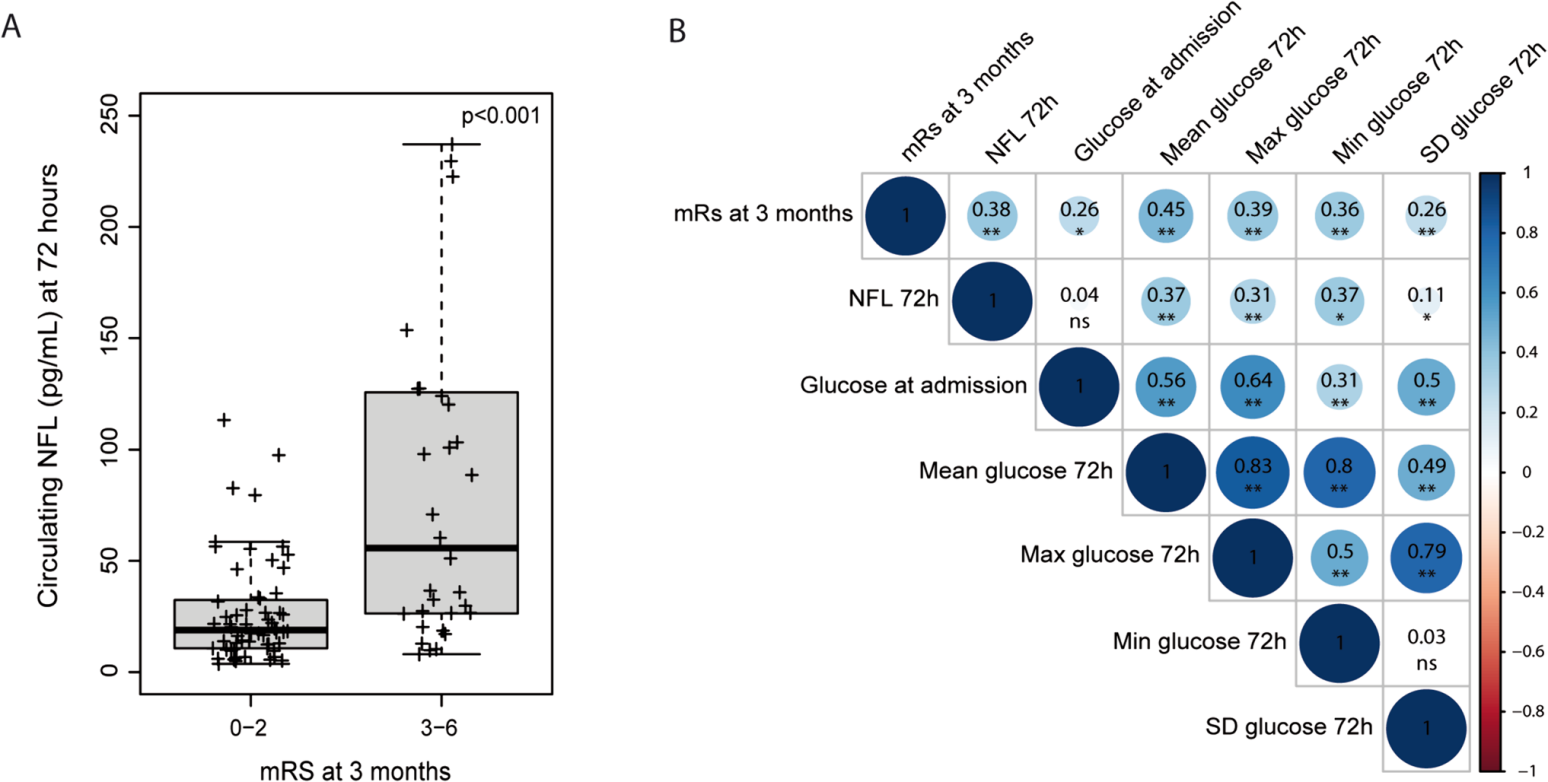
**A** NFL levels at 72 h after admission in patients with good (mRS 0–2) and poor (mRS 3–6) outcome at 3 months. **B** Correlation matrix between mean glucose in 72 h, NFL levels at 72 h, and poor clinical outcome at 3 months. Numerical data within the matrix represent Spearman’s rank correlation coefficients; single asterisk means *p* value lower than 0.05; double asterisks mean *p* value lower than 0.01. NFL, neurofilament light chain; mRS, modified Rankin Scale; SD, standard deviation

The median (IQR) levels of NFL at the end of the EBI period were 25 (13–56) pg/mL. The variables associated with higher NFL levels in univariate analysis included older age, poorer initial WFNS grade, and higher mean glucose levels during the EBI period, as shown in Table 4. In multivariate analyses, only glucose levels remained associated with NFL [adjusted OR 1.01 (95% CI 1.001–1.024, *p* = 0.03)]. The levels of NFL were significantly higher in patients with poor clinical outcome [median (IQR) 56 (26–126) versus 19 (11–32) in patients with poor and good clinical outcome, respectively; *p* < 0.001], as shown in Fig. 1A. A matrix of correlations between glucose metrics assessed during the EBI period, NFL levels measured at 72 h, and mRS at 3 months is shown in Fig. 1B. Of note, the association between NFL levels and poor clinical outcome remained significant in binary regression models adjusted by age, pre-morbid mRS, WFNS grade at hospital admission, and mean glucose levels during the EBI period [adjusted OR 2.61 (95% CI 1.364–5.010, *p* = 0.004)].

**Table 4:** Univariate and multivariate analyses of the associations between serum NFL levels (log-transformed) and baseline variables

Variables	Univariate analysis OR (95% CI), *p* value	Multivariate analysis OR (95% CI), *p* value
Age (per year)	1.02 (1.000–1.034), *p* = 0.045	1.01 (0.996–1.027), *p* = 0.162
Female sex (vs male)	1.22 (0.765–1.928), *p* = 0.409	-
Pre-morbid mRS (per IQR)	1.17 (0.874–1.575), *p* = 0.288	-
Hypertension (yes)	1.27 (0.814–1.972), *p* = 0.294	-
Dyslipidemia (yes)	1.52 (0.867–2.646), *p* = 0.145	-
WFNS grade (4–5)	2.11 (1.385–3.222), *p* = 0.001	1.51 (0.926–2.463), *p* = 0.098
Aneurysm (yes)	1.52 (0.701–3.287), *p* = 0.290	-
Aneurysm size (per IQR)	1.13 (0.925–1.379), *p* = 0.233	-
Modified Fisher Scale (per IQR)	1.10 (0.862–1.399), *p* = 0.448	-
Intraventricular bleeding (yes)	1.09 (0.659–1.786), *p* = 0.750	-
Hydrocephalus (yes)	1.21 (0.770–1.902), *p* = 0.408	-
Parenchymal hemorrhage (yes)	1.61 (0.970–2.661), *p* = 0.065	1.34 (0.834–2.151), *p* = 0.227
Global cerebral edema (yes)	1.08 (0.696–1.688), *p* = 0.722	-
Aneurysm treatment modality (per type)	1.03 (0.757–1.397), *p* = 0.858	-
Mean glucose within 72 h (per mg/dL)	1.02 (1.011–1.030), *p* < 0.001	1.01 (1.001–1.024), *p* = 0.039

*OR* odds ratio, *CI* confidence interval, *mRS* modified Rankin Scale, *IQR* interquartile range, *WFNS* World Federation of Neurosurgical Societies Scale

### Glucose Levels During the EBI Period, Circulating NFL Levels at the End of the EBI Period, and Clinical Outcome at 90 Days: A Mediation Analysis

**Fig. 2:**
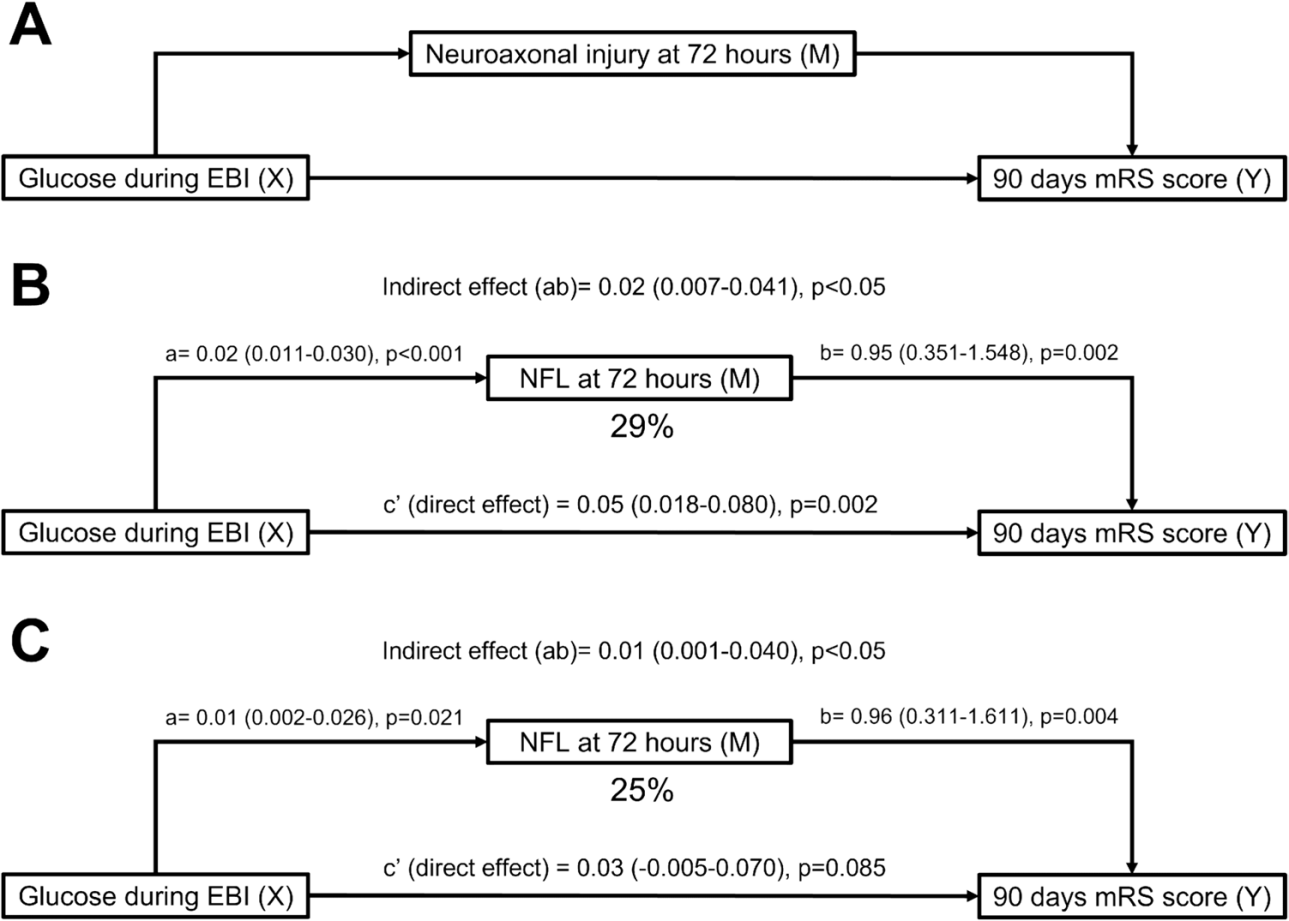
Model of the mediation pathways between glucose levels within the first 72 h after bleeding onset (glucose during EBI) and clinical outcome at day 90 according to circulating NFL levels (log-transformed) measured at 72 h after bleeding (NFL at 72 h). **A** Theoretical framework of a pathway model designed to explain the mediation effect of NFL on the association between glucose and clinical outcome at day 90 (dichotomized mRS score; poor outcome defined as mRS > 2). **B**, **C** Unadjusted and adjusted path mediation analyses, respectively. Coefficients in the path models are listed as a, b, and c′, where a and b are part of the indirect path and c′ is the direct path adjusted for the indirect path; the total effect is the composition of the direct effect (c′) and the indirect effect (ab). Data are coefficient estimates with 95% confidence intervals. EBI, early brain injury; NFL, neurofilament light chain; mRS, modified Rankin Scale; M, mediator

A mediation analysis was implemented to evaluate whether the association between higher glucose levels during the EBI period and clinical outcome was mediated through its association with increased NFL levels, as schematically shown in Fig. 2A. As shown in Fig. 2B, in unadjusted analyses, glucose levels during EBI were associated with higher circulating NFL levels at 72 h (mediator variable), and both glucose and NFL levels were also significantly associated with poor clinical outcome. Mediation analysis between glucose levels and poor clinical outcome at 90 days revealed a significant indirect effect (coefficient = 0.02, 95% CI = 0.007–0.041, *p* < 0.005) indicating that the association between glucose and clinical outcome was in part mediated by NFL levels. After adjustment for the mediator NFL, glucose still had a substantial association with functional outcome. Overall, in unadjusted analyses, the mediator NFL explained 29% of the association of higher glucose with poor functional outcome. In analyses adjusted by age, WFNS at hospital admission and pre-morbid mRS, the associations between glucose, NFL, and clinical outcome remained significant, as shown in Fig. 2C. Adjusted mediation analyses revealed no significant direct effect of glucose on functional outcome, while there was a significant indirect effect that was mediated by NFL levels (coefficient = 0.01, 95% CI = 0.001–0.040, *p* < 0.005). Overall, in adjusted analyses, the mediator NFL explained 25% of the association of higher glucose levels with poor functional outcome.

## Discussion

In agreement with previous reports, in this cohort of SAH patients, higher glucose levels during the EBI period were associated with poor clinical outcome at long term. Acute hyperglycemia during the EBI period also predicted higher circulating NFL levels measured at the end of the EBI period. According to mediation analyses, the association between glucose and poor clinical outcome was significantly mediated through the link between glucose and NFL levels. These observations suggest that the association between hyperglycemia and poor clinical outcome in SAH might be explained in part through glucose-driven secondary NAD.

In line with current knowledge, in this cohort, glucose levels measured during the EBI period after SAH onset emerged as one of the most robust predictors of poor outcome, along with increased clinical severity at hospital admission [15–20]. In our sample, all the explored glucose metrics except the standard deviation were associated with clinical outcome at 90 days. According to ROC analysis, mean glucose levels during the EBI period disclosed the highest accuracy for predicting poor clinical outcome at 3 months, especially in comparison with admission glucose levels. This observation is in line with previous reports. Nonetheless, we found no association between standard deviation and prognosis in our cohort, whereas there is increasing evidence that dynamic glucose parameters during the first days after stroke could be more informative for the prediction of in hospital complications or long-term clinical outcome than isolated initial measurements [16, 17, 38–42]. This could be explained due to the limited temporal resolution of glucose metrics derived from current standards of care, thus limiting the power of assessments regarding glucose dynamics and prognosis. In this context, the use of comprehensive dynamic evaluations such as those derived from continuous glucose monitoring devices could aid to obtain a more precise knowledge on the prognostic and physiopathological relevance of glycemic patterns during the acute phase of SAH [42].

In this study, we evaluated the presence of NAD non-invasively through the measurement of circulating NFL levels at the end of the EBI period. Therefore, circulating NFL levels were used as a surrogate marker or consequence of ongoing brain injury during the EBI period and not as a driver (or direct cause) of poor clinical outcome at long term. Up to less than a decade ago, NFL could only be reliably measurable in CSF due to technical limitations. The development of the Simoa technique allows NFL measurement in peripheral biological samples, then making possible an in-depth evaluation of NFL as a biomarker of disease severity and prognosis. According to previous reports, in the setting of SAH, higher NFL levels correlate consistently with worse clinical and radiological severity of the bleeding at admission as well as with poor clinical outcome at long term [25–33]. In our cohort, we confirmed the association between serum NFL levels and poor clinical outcome at 3 months after bleeding. As a novel finding, we found that higher serum NFL levels measured at the end of the EBI period were predicted by higher glucose levels measured during the acute phase after bleeding in adjusted linear regression analysis, independently of age or clinical status at hospital admission. Given the significant multilateral associations found between glucose levels during the EBI period, NFL levels measured at the end of the EBI period, and clinical outcome at 90 days, we implemented a mediation analysis to explore whether the association between glucose and poor clinical outcome was mediated through NFL levels as a mediator variable. According to these analyses, the association of glucose with NFL levels was stronger than its direct association with clinical outcome, thus suggesting that the effect of glucose on long-term clinical outcome was at least in part mediated by the magnitude of neuroaxonal injury. Indeed, the indirect mediation pathway explained between 29 and 25% of the association between glucose and clinical outcome at day 90 in unadjusted and adjusted mediation analyses, respectively. Overall, these data suggest that elevated glucose levels during the EBI period might hamper the clinical recovery at long term by promoting glucose-driven neuroaxonal injury. This supports the conceptualization of glucose as an active agent in neuronal damage rather than a passive epiphenomenon of disease severity. However, as mediation analyses do not imply causality, the validity and direction of these relationships deserve further investigation in additional larger prospective observational cohort studies and in interventional preclinical and clinical trials. According to our findings, the use of surrogate markers of NAD such as NFL levels could be valuable for the assessment of the biological effect of interventional trials aimed to improve glycemic control or specific pharmacological agents.

The main strength of this study was the use of a well-characterized prospective cohort of consecutive SAH subjects admitted in a Comprehensive Stroke Center. Furthermore, NFL levels were assessed through a well-validated fourth generation Simoa technique. Also, the mediation analysis approach, even if no causation is implied, sheds light on the potential glucose-driven harmful pathways triggered during EBI period. Nevertheless, this study has several limitations. First, we used unadjusted and adjusted mediation analyses to characterize of the association between hyperglycemia, NAD, and clinical outcome in our cohort of SAH patients. Given the observational nature of this study, the estimated direct and indirect effects of the association between hyperglycemia and clinical outcome may have been affected by unmeasured confounding. Therefore, the mediation effect found in this study should be interpreted with caution and requires further confirmation in external observational cohorts and in interventional studies [43]. Second, NFL levels were assessed at the end of the EBI period as a surrogate biomarker of brain injury to reflect the amount of NAD occurring during the whole EBI period. Consequently, our results might be only applicable to the subset of SAH patients who survive beyond 72 h after bleeding onset. Third, this was an observational study where hyperglycemia was managed according to local in-hospital protocols and therefore a cause-and-effect relationship cannot be inferred from the obtained data. Fourth, NAD was exclusively evaluated using one single blood biomarker in a single time point. The assessment of longitudinal serum NFL levels and/or additional NAD surrogate biomarkers such as those derived from advanced quantitative MRI including microvascular, metabolic, and microstructural integrity measurements could be highly informative for identifying glucose profiles associated with higher brain damage after SAH. A better knowledge on the link between advanced quantitative neuroimaging metrics or NFL levels and longitudinal glucose profiles during the EBI period is needed to understand the most appropriate and informative timing and how to combine the different surrogate markers of acute brain injury. Eventually, this information may lead to improve glucose management protocols and to optimize the design of clinical trials aimed to modulate glucose levels in the acute phase after SAH. Finally, this report lacks additional mechanistic studies on molecular levels to explain how high glucose levels lead to increased NFL levels. Further preclinical and clinical studies are needed to confirm this relationship and to understand the mechanistic link between glucose disturbances and neuroaxonal damage in this disease.

## Conclusions

Both higher mean glucose measured along the EBI period and higher NFL levels measured at the end of the EBI period were independently associated with poor clinical outcome at 90 days. The mediation analyses suggested that approximately 25% of the variability of the association between glucose and poor prognosis could be mediated through neuroaxonal damage as measured via circulating NFL levels. These results suggest that the link between hyperglycemia and poor clinical outcome may be partly explained by secondary neuroaxonal injury. Additional research is needed to confirm these associations and to explore the mechanistic link between hyperglycemia and secondary brain damage.

## Supplementary Information

Below is the link to the electronic supplementary material.

Supplementary file1 (DOCX 24 KB)

## Data Availability

The datasets generated and analyzed are available from the corresponding authors on reasonable request.
