# Supplementary material for: Neuroaxonal Injury May Mediate the Association Between Hyperglycemia and Prognosis in Spontaneous Subarachnoid Hemorrhage
Source: Mol Neurobiol. 2024 Jul 12;62(2):1467–77. doi: 10.1007/s12035-024-04347-6 (PMC11772382; doi:10.1007/s12035-024-04347-6)
Supplement: Supplementary file 1 — Supplementary file1 (DOCX 24 KB) [file 12035_2024_4347_MOESM1_ESM.docx]

^pSAH: Perimesencephalic Subarachnoid Haemorrhage. DM: Diabetes Mellitus. mRS: Modified Rankin Scale.^

**Fig.S1** Schematic flow chart of patient inclusion and follow up. Study period from 27^th^ September, 2018, to 1^st^ June, 2021. Of note, 7 patients with pSAH pattern also presented a previous history of DM
